# *Bolbitis lianhuachihensis* (Dryopteridaceae), a new species from Taiwan

**DOI:** 10.3897/phytokeys.131.36548

**Published:** 2019-09-05

**Authors:** Yi-Shan Chao, Yu-Fang Huang, Shi-Yong Dong, Yao-Moan Huang, Ho-Yih Liu

**Affiliations:** 1 Department of Biomedical Science & Environmental Biology, Kaohsiung Medical University, 100, Shih-Chuan 1st Rd., Kaohsiung, 80708, Taiwan Kaohsiung Medical University Kaohsiung Taiwan; 2 Department of Biological Sciences, National Sun Yat-sen University, Kaohsiung 80424, Taiwan National Sun Yat-sen University Kaohsiung Taiwan; 3 South China Botanical Garden, Chinese Academy of Sciences, Guangzhou 510650, China South China Botanical Garden, Chinese Academy of Sciences Guangzhou China; 4 Taiwan Forestry Research Institute, 53 Nan-Hai Rd., Taipei, 10066, Taiwan Taiwan Forestry Research Institute Taipei Taiwan

**Keywords:** Ferns, Taiwan, taxonomy, venation

## Abstract

A new species of *Bolbitis*, *B. lianhuachihensis***sp. nov.**, was found in central Taiwan. It most resembles B. virens var. compacta and *B. hainanensis*. A phylogenetic tree of Taiwanese and other Asian species of *Bolbitis* species supports the recognition of the new species. Morphologically, the combination of anastomosing venation and fewer sterile pinnae are critical characters to discriminate *B. lianhuachihensis* from other Taiwanese *Bolbitis* species. *Bolbitis lianhuachihensis* can be further distinguished from B. virens var. compacta and *B. hainanensis* by having lanceolate sterile pinnae and absent or fewer free veinlets in small areoles of sterile pinnae. The morphological descriptions, illustration, ecology and distribution of the new species are presented. A key to all Taiwanese *Bolbitis* is also provided.

## Introduction

*Bolbitis* Schott is a pantropical genus that belongs to Dryopteridaceae and consists of about 80 species (Moran et al. 2010a; PPGI 2016). The plants are terrestrial or lithophytic and usually grow in damp forests, such as valleys or in and along streams. Most *Bolbitis* species have proliferous buds on the terminal pinnae near their apices (Hennipman 1977; Moran et al. 2010b). The genus also exhibits strong sterile-fertile frond dimorphism, and most of its species are pinnate.

In Taiwan, ten taxa have been recorded; namely, *B. angustipinna* (Hayata) H.Ito, *B. appendiculata* (Willd.) K.Iwats., *B. heteroclita* (C.Presl) Ching, *B. laxireticulata* K.Iwats., B. × nanjenensis C.M.Kuo, *B. rhizophylla* (Kaulf.) Hennipman, *B. scalpturata* (Fée) Ching, *B. subcordata* (Copel.) Ching, B. virens (Wall. ex Hook. & Grev.) Schott var. compacta Hennipman and *B. heteroclita* × *B. subcordata* (Tsai and Shieh 1994; Knapp 2011; 2013). Three of these taxa, *B. laxireticulata*, B. × nanjenensis and *B. heteroclita* × *B. subcordata* are presumed to have a hybrid origin (Iwatsuki 1959; Kuo 1990; Knapp 2011).

**Figure 1.**
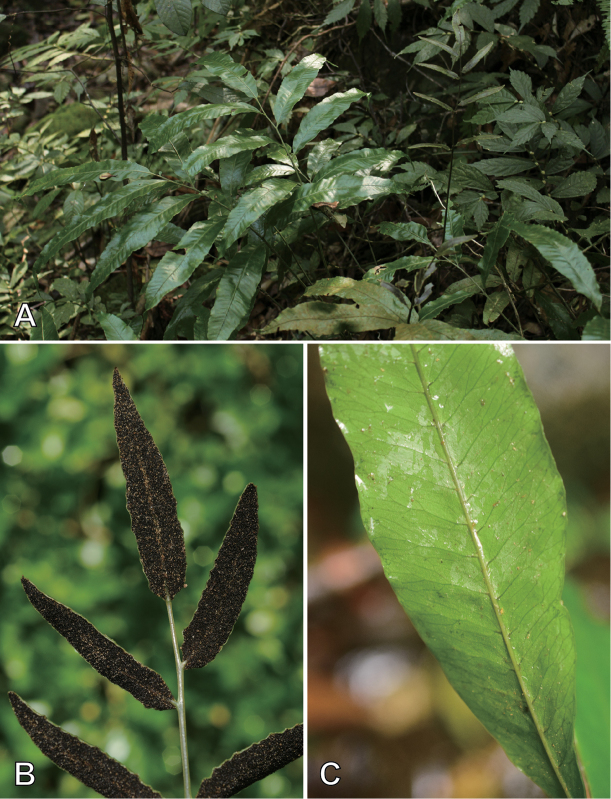
*Bolbitis lianhuachihensis***A** habit; note taller fertile fronds (photographed by Y.-S. Chao) **B** acrostichoid sori (photographed by P.-F. Lu) **C** venation of sterile frond (photographed by Y.-F. Huang).

In Taiwan, a *Bolbitis* plant appeared unusual due to its few sterile pinnae (Figs 1 and Suppl. material 1: Figure S1). By this character, it was identified as B. virens var. compacta and resembled *B. hainanensis* Ching & Chu H. Wang (Knapp 2011), but B. virens var. compacta is found in Indochina (Hennipman 1977), while *B. hainanensis* is endemic to Hainan and Yunnan, China (Dong and Zhang 2005; Zhang et al. 2013). Taiwanese *Bolbitis* also differed from these species by venation. This character has been shown to be helpful in distinguishing many species of *Bolbitis* worldwide (Hennipman 1977). Species of *Bolbitis* may have either free or anastomosing veins. If the latter, a helpful distinguishing character is the number of areoles between the costae and margins, the presence or absence of included free veinlets in the areoles and whether these veinlets are recurrent or excurrent (Hennipman 1977; Moran et al. 2010a). In this study, we compared the morphological characteristics of the undescribed plant, especially with regards to lamina venation, with other species of *Bolbitis* in Taiwan. We also examined the phylogenetic relationship of these plants to existing *Bolbitis* species in Taiwan and to other similar species. Based on the results, we were able to clarify the specific morphological and molecular traits of the *Bolbitis* plant and describe a new species.

## Materials and methods

### Morphological studies

The undescribed *Bolbitis* was collected in central Taiwan and herbarium specimens at HAST and TAIF were also studied. The keys, descriptions and illustrations in Hennipman’s monograph (1977) were investigated to compare the morphological characteristics of our suspected new species with the known *Bolbitis* species. Furthermore, the type specimens of two similar species in nearby regions, *B. hainanensis* (*Wallich 1033*, K) and B. virens var. compacta (*Tagawa et al. 6802*, L) were analysed. As the type specimen of *B. hainanensis* lacks fertile fronds, we also consulted the protologue (Ching and Wang 1983). The venation of the Lienhuachih plants, two similar taxa and the known *Bolbitis* taxa in Taiwan were depicted. The morphological terminology follows that of Lellinger (2002). Acronyms of herbaria follow Thiers (2019).

### Phylogenetic analyses

To clarify the phylogenetic position of the undescribed plant, six more *Bolbitis* species from Taiwan and *B. virens* from China were sampled and sequenced in this study. Additional sequences of 19 species were gathered from GenBank, including 17 *Bolbitis* species and two outgroup species. *Elaphoglossum lepervanchii* and *Teratophyllum koordersii* were used as outgroups, based on the phylogenetic trees of *Bolbitis* and related taxa (Moran et al. 2010a; Chen et al. 2017). A total of 30 samples of *Bolbitis* species were included and the voucher information is provided in the Appendix 1.

Total genomic DNA was extracted from young fronds, following a modified cetyltrimethylammonium bromide (CTAB) method (Doyle and Doyle 1990). Two chloroplast markers were used: *rps4-trnS* intergenic spacer was amplified by the primers *rps4-3r.f* (AGT TGT TAG TTG TTG AGT AT) (Skog et al. 2004) and *rps4-trnS* (TAC CGA GGG TTC GAA TC) (Souza-Chies et al. 1997); *trnL*-*F* intergenic spacer was amplified by the primers from Taberlet et al. (1991) (primer e – GGT TCA AGT CCC TCT ATC CC and primer f – ATT TGA ACT GGT GAC ACG AG). All sequences were aligned using ClustalW (Thompson et al. 1994) and then were manually edited using BioEdit 7.1.3 (Hall 1999). Gaps were treated as missing data.

Phylogeny was inferred by Maximum Likelihood (ML) analyses with GARLI v.2.0.1019 (Zwickl 2006). The best tree was created from the ten independent runs with automatic termination following 10,000 generations without a significant (lnL increase of 0.01) change in topology. A majority-rule consensus tree was calculated in PAUP* v. 4.0b10 (Swofford 2002) to obtain bootstrap support based on 1,000 bootstrap replicates with automatic termination at 10,000 generations under one run in GARLI. Genetic data and the accession numbers of the sequences are listed in the Appendix 1.

## Results

Based on the morphological study of the undescribed plant and type specimens of *Bolbitis hainanensis* and B. virens var. compacta (Suppl. material 1: Figure S2 and S3, respectively), specific characteristics were analysed to separate the three taxa; morphological data in the *Bolbitis* monograph (Hennipman 1977) is also integrated in Table 1. The unknown plant is distinguished from the two morphologically similar species through several characters: smallest fertile pinnae, lanceolate sterile pinna (narrower than others), cuneate bases of sterile pinnae, the smallest angle between veinlets and costae of sterile pinnae and the absence of, or fewer, free veinlets in small areoles of sterile fronds.

**Table 1.** Morphological comparisons amongst *Bolbitis lianhuachihensis*, *B. hainanensis* and B. virens var. compacta.

Characters	*B. lianhuachihensis*	*B. hainanensis*	B. virens var. compacta
Texture of laminae	Chartaceous	Coriaceous	Chartaceous
Size of fertile pinnae	5–9 × 0.8–1.2 cm	6–10 × 1–1.5 cm	4–11.5 × 0.8–2 cm
Number of lateral pinnae of sterile fronds	1–5 pairs	2–3 pairs	2–7 pairs
Sterile pinna shape	Lanceolate	Oblong-lanceolate	Lanceate
Size of sterile pinnae	14–24 × 3–4.5 cm	17–22 × 5–6 cm	8–23 × 3–4 cm
Bases of sterile pinnae	Cuneate	Narrowly cuneate	Narrowly cuneate or obtuse
Margins of sterile pinnae	Entire	Entire or toothed	Entire
Veinlets with an angle to costae of sterile pinnae	ca. 65°	75°–80°	75°–80°
Row number of areoles between the costae and margins in sterile fronds	2–4 rows	3–4 rows	4–5 rows
Free veinlets in areoles of sterile fronds	Absent or very few	Present	Present

**Figure 2.**
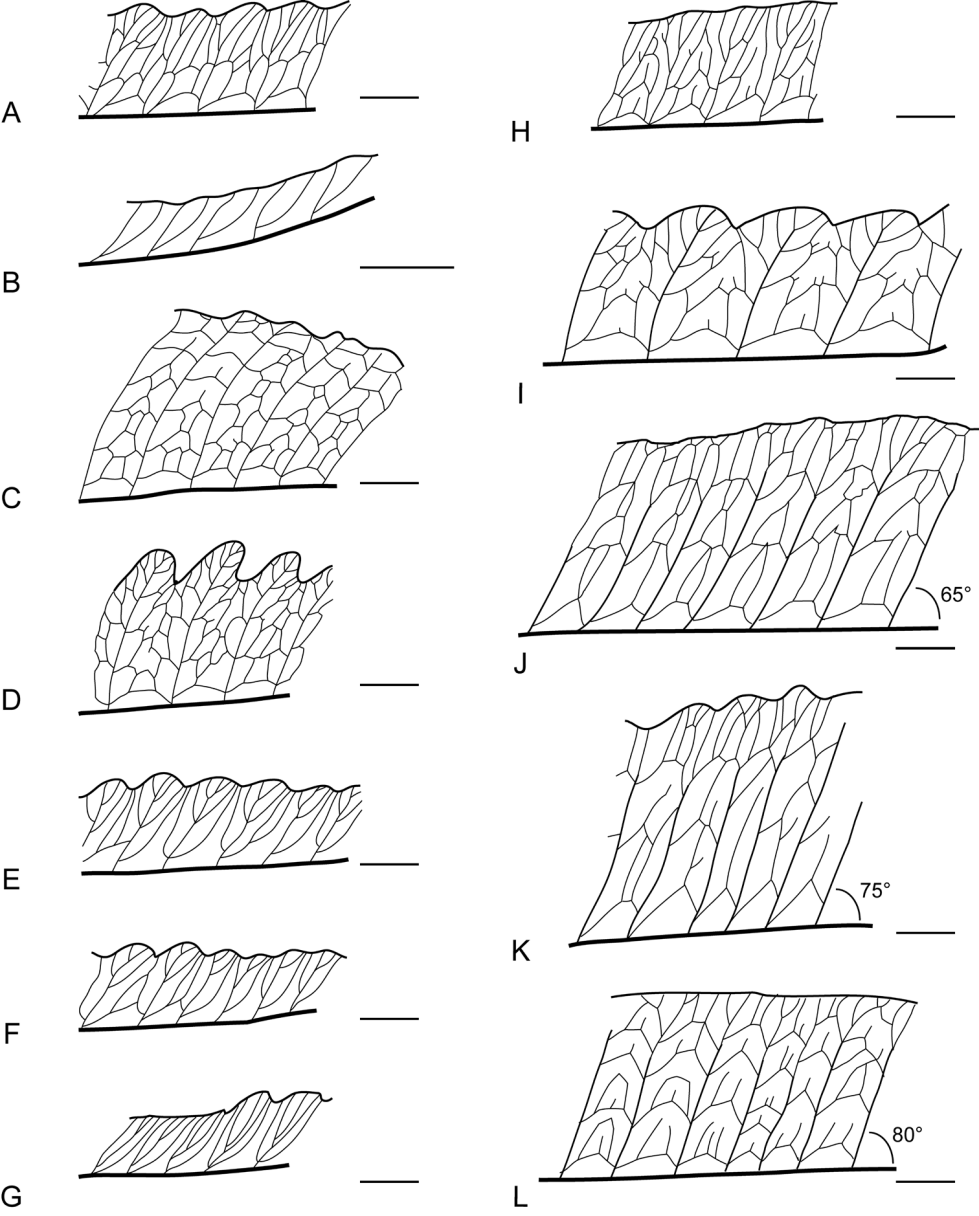
Venation patterns of sterile pinnae from all species of *Bolbitis* in Taiwan **A***Bolbitis angustipinna* (*Y.-F. Huang 176*, TAIF) **B***Bolbitis appendiculata* (*Y.-F. Huang* 243, TAIF) **C***Bolbitis heteroclita* (*Y.-F. Huang* 221, TAIF) **D**Bolbitis heteroclita × subcordata (*Y.-F. Huang* 366, TAIF) **E***Bolbitis laxireticulata* (*Y.-F. Huang* 228, TAIF) **F**Bolbitis × nanjenensis (*Y.-F. Huang* 213, TAIF) **G***Bolbitis rhizophylla* (*Y.-F. Huang 124*, TAIF) **H***Bolbitis scalpturata* (*Y.-F. Huang* 164, TAIF) **I***Bolbitis subcordata* (*Y.-F. Huang* 254, TAIF) **J***Bolbitis lianhuachihensis* (holotype specimen, *Y.-S. Chao 3006*, TAIF) **K***Bolbitis hainanensis* (holotype specimen, *C. Wang 35870*, PE) **L**Bolbitis virens var. compacta (type specimen, *Tagawa et al. 6802*, L). The angles of veinlets to costae are indicated in *B. lianhuachihensis* (**J**), *B. hainanensis* (**K**), and B. virens var. compacta (**L**). Scale bars: 5 mm.

The venation in sterile fronds of the unknown plant, *Bolbitis hainanensis*, B. virens var. compacta and other *Bolbitis* species in Taiwan is illustrated in Fig. 2, showing one side of a pinna of each species. We have found that the venation pattern of some taxa vary between small and larger pinnae, which is also reported by Hennipman (1977). Here we studied the larger pinnae of each taxon. Based on the venation patterns, the examined *Bolbitis* taxa can be divided into three groups: *Bolbitis appendiculata* and *B. rhizophylla* have free veins; *B. laxireticulata* and B. × nanjenensis have mostly free veins and few anastomosing veins; the other species have anastomosing veins. The species with anastomosing veins can be further classified by free veinlets included in areoles or not. *Bolbitis hainanensis*, *B. heteroclita*, *B. scalpturata*, *B. subcordata* and B. virens var. compacta have free veinlets included in areoles; *B. angustipinna* and the undescribed plant (in pinnae wider than 3.5 cm) have no or very few free veinlets; *B. heteroclita* × *B. subcordata* have few free veinlets. Furthermore, the number of the areole rows between the costae and margins in sterile fronds varies amongst the species. *Bolbitis angustipinna*, *B. scalpturata* and *B. subcordata* have 2–3 rows; *B. hainanensis* and *B. heteroclita* × *B. subcordata* have 3–4 rows; B. virens var. compacta and the unknown plant have 4–5 rows; *B. heteroclita* has more than 5 rows.

We also find the angles of veinlets to costae to vary. Considering the three similar taxa, the unknown plant has smaller angles than *B. hainanensis* and B. virens var. compacta (65°< 75° or 80°; Fig. 2 J, K, and L); the latter two taxa have sterile pinnae with veinlets more vertical to the costae than the unknown species (also in the type specimens of the three taxa Suppl. material 1: Figures S1, S2, and S3).

Three presumed hybrid taxa present intermediate venation morphology between their putative parents. *Bolbitis heteroclita* × *B. subcordata* has more free veinlets included in areoles than *B. heteroclita*, but fewer free veinlets included in areoles than *B. subcordata*. Most pinnae of *B. laxireticulata*, like *B. appendiculata*, have free veins, but some pinnae have anastomosing veins, similar to those of *B. subcordata*. Some pinnae of Bolbitis × nanjenensis have free veins like *B. appendiculata*, but some have costal areoles like *B. heteroclita*.

### Molecular phylogenetic analyses

**Figure 3.**
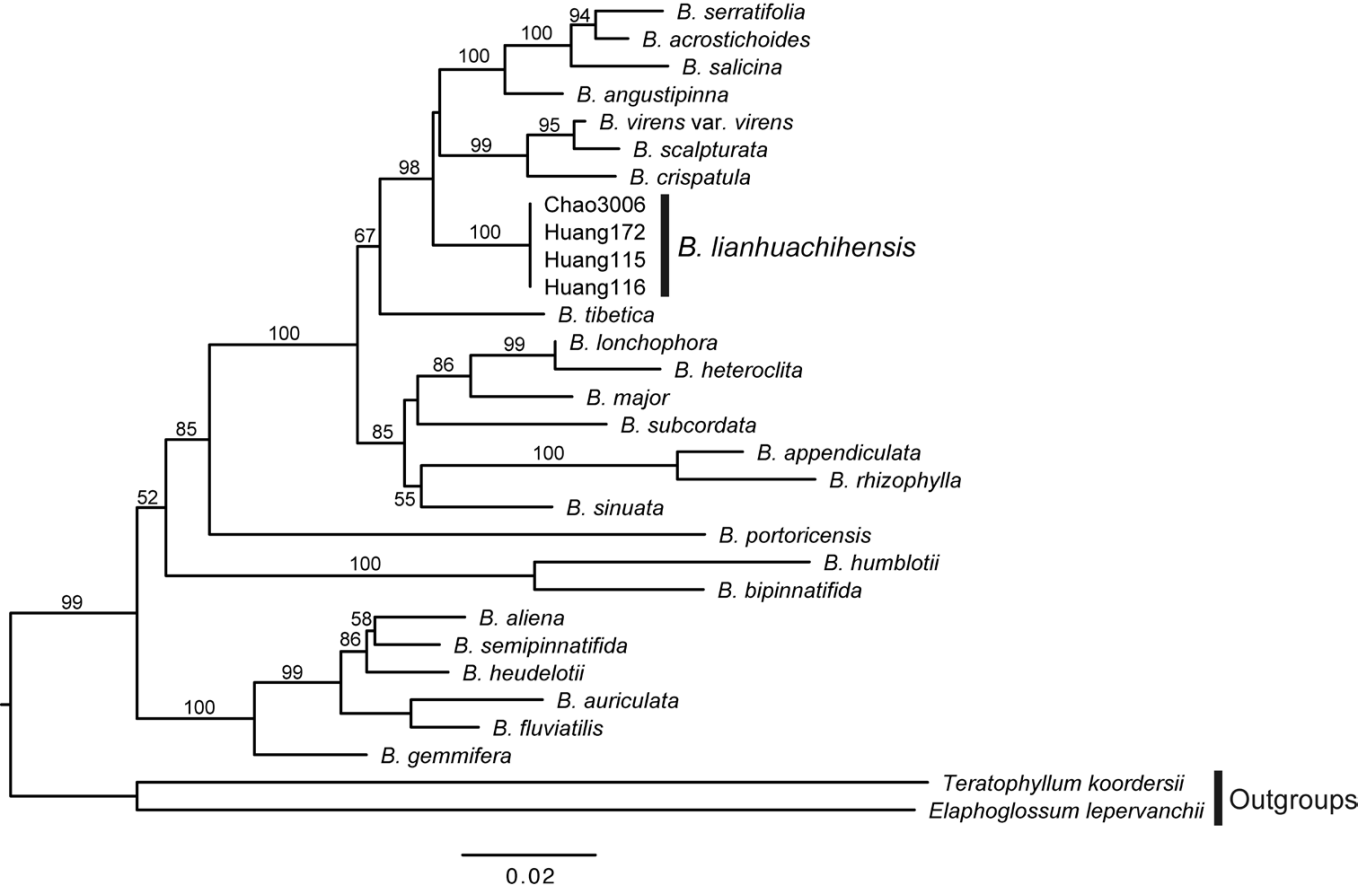
Chloroplast DNA phylogeny of 25 *Bolbitis* species and two outgroups. ML bootstrap support values are indicated on each branch.

The chloroplast DNA (cpDNA) alignment of *rps4-trnS* and *trnL-F* contained 549 bp and 428 bp, respectively, with 239 parsimony-informative sites in total. The log-likelihood score for the most likely ML tree was -4989.8277. Our four specimens of Lienhuachih *Bolbitis* shared the same genotype and occupied a unique place that was well-separated from all other samples species in the phylogenetic tree, different from other Taiwanese *Bolbitis* species and B. virens var. virens (Fig. 3). In contrast to *B. heteroclita* and *B. subcordata*, the undescribed plant and B. virens var. virens are in the same clade with unresolved subclades. Bolbitis virens var. virens and *B. scalpturata* are sister species, then they clustered with *B. crispatula*; the undescribed plant is more phylogenetically distant to B. virens var. virens.

### Taxonomic treatment

#### Bolbitis lianhuachihensis

Taxon classificationPlantaePolypodialesDryopteridaceae

Y.S.Chao, Y.F.Huang, & H.Y.Liu sp. nov.

DA4F28BC607A5FE28156C1F219A0C87A

urn:lsid:ipni.org:names:77201650-1

Fig. 1 and Suppl. material 1: Figures S1

Bolbitis virens var. compacta auct. non Hennipman: Knapp, Ferns Fern Allies Taiwan: 440. 2011.

##### Type.

TAIWAN. Nantou County: Lienhuachih, 700 m a.s.l., 11 March 2018, *Yi-Shan Chao 3006* (holotype TAIF!, isotype TAIF!).

***Bolbitis lianhuachihensis*** morphologically resembles B. virens var. compacta and *B. hainanensis*, from which it is distinguished in having lanceolate sterile pinnae and no free veinlets in areoles of sterile fronds.

##### Description.

Terrestrial or lithophytic. Rhizome short-creeping, thick, densely scaly; scales concolorous, black, lanceolate, 3–5 mm long, 0.5 mm wide, margin entire. Fronds clustered, 40–80 cm long, dimorphic, pinnate. Sterile fronds with stipes 18–50 cm long, near base 2–4 mm diam., scaly, glabrous upwards; lamina broad-ovate, 21–34 × 21–35 cm, chartaceous, conform; lateral pinnae 1–5 pairs, alternate, lanceolate, 14–24 × 3–4.5 cm, bases cuneate, margins entire, undulate, apices caudate or acuminate, basal two pairs of pinnae with winged petiolules < 8 mm; terminal pinna larger or similar to lateral pinnae, sometimes with a bulbil near the apex; veins reticulate, 2–4 rows, no or very few areoles with free veinlets in the largest pinnae (wider than 3.5 cm). Fertile fronds longer than or as long as the sterile ones; stipes 35–50 cm long; lamina oblong-ovate, 7.5–14.5 × 12–18 cm; pinnae 3–4 pairs, alternate, lanceolate, 5–7.5 × 0.8–1.2 cm, base narrow-cuneate, apex acuminate, stalked. Sporangia acrostichoid.

##### Additional specimens examined.

**TAIWAN. Chiayi County**: Tsenwen Dam, 24 Jan. 1987, *Bi-Jao Wang 10039*, *11009* (HAST, TAIF). **Nantou County**: Lienhuachih, 700 m a.s.l., *Yih-Han Chang 20070317-008* (TAIF), 29 Aug. 2009, *Cheng-Wei Chen Wade 955* (TAIF), 22 Mar. 2015, *Cheng-Wei Chen Wade 4181* (TAIF), 29 Jan. 2016, *Yu-Fang Huang 115*, *116*, *117* (TAIF), 10 Oct. 2005, *Ralf Knapp 697* (P), 22 Oct. 2011, *Ralf Knapp 20111022-4* (HAST), 4 Dec. 2012, *Pi-Fong Lu 24940* (TAIF), 23 Apr. 2006, *Wei-Hsiu Wu s.n.* (TAIF); Tiandi, 830 m a.s.l., 8 Aug. 2006, *Ralf Knapp 20060806-18* (HAST, TAIF), 900 m a.s.l., 26 Sep. 2016, *Yu-Fang Huang 172*, *173*, *174* (TAIF).

##### Distribution.

Taiwan (Fig. 4).

**Figure 4.**
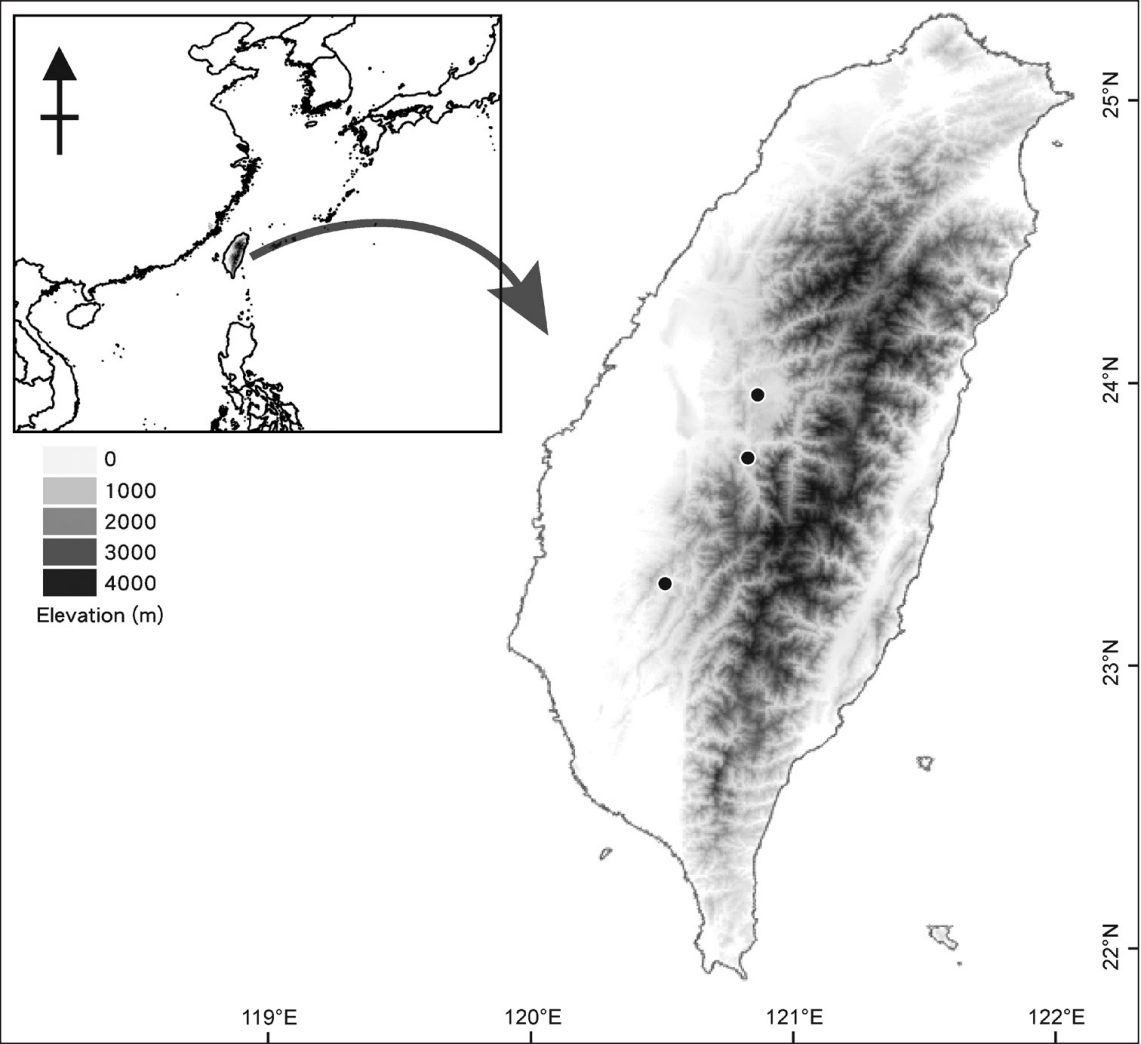
Distribution of *Bolbitis lianhuachihensis* (black circles) in Taiwan.

##### Ecology.

Evergreen, broad-leaf forests, often near streams, below 1000 m a.s.l.

##### Etymology.

The specific epithet ‘lianhuachihensis’ refers to the type locality.

##### Common name (assigned here).

Lian Hua Chih Shih Jyue (蓮華池實蕨; Chinese name).

##### Preliminary conservation assessment.

To date, only three small populations of *Bolbitis lianhuachihensis* Y.S.Chao, Y.F.Huang, & H.Y.Liu have been recorded in Taiwan. However, the estimated number of individuals is smaller than 250. It meets the category Endangered (EN D1) based on the IUCN (2017) criteria.

##### Note.

*Bolbitis lianhuachihensis* can be delimited by the combination of anastomosing venation and fewer sterile pinnae than any other species of *Bolbitis* in Taiwan. It was thought to be related to two similar taxa with “thick laminae” in nearby regions, B. virens var. compacta and *B. hainanensis* (Knapp 2011). However, based on our study, only *B. hainanensis* has coriaceous laminae, thicker than chartaceous laminar of *B. lianhuachihensis* and B. virens var. compacta. Moreover, *B. lianhuachihensis* has lanceolate sterile pinnae and very few free veinlets in areoles of sterile pinnae wider than 3.5 cm, differing from the two similar taxa.

In this study, we revealed the venation diversity in the Taiwanese *Bolbitis* species and related taxa. Based on the illustration of venation, it is found that every taxon possesses its own venation morphology, supporting the taxonomic value of venation (Hennipman 1977; Moran et al. 2010a). We have applied several characters, including angles of veinlets to costae, free or anastomosing, row number of areoles between the costae and margins in sterile fronds and free veinlets in areoles or not for sterile fronds. We also found that venation patterns are more complicated, composed of multiple areoles, sub-areoles and free veinlets, which is also reported by Hennipman (1977). A character-state change from free venation to anastomosing venation is reported by Moran et al. (2010a). The venation characteristics in *Bolbitis* species are worthy of further investigation and application to the systematic and evolutionary study. To classify and describe the diverse venation morphology in detail would contribute to further studies of *Bolbitis* systematics.

### Key to *Bolbitis* species and hybrids in Taiwan

**Table:** 

1	Veins free	**2**
–	Veins anastomosing	**3**
2	Sterile pinnae unequal at base, auriculate; pinna margins with sharp teeth in sinuses	***B. appendiculata***
–	Sterile pinnae equal at base; pinna margins without sharp teeth	***B. rhizophylla***
3	Sterile fronds with few areoles, sometimes only a single series of costal areoles	**4**
–	Sterile fronds with many areoles, more than one row of areoles between the costae and margins	**5**
4	Sterile pinnae linear-lanceolate	***B. laxireticulata***
–	Sterile pinnae falcate	**B. × nanjenensis**
5	Sterile pinnae linear-lanceolate	6
–	Sterile pinnae lanceolate	8
6	Sterile lateral pinnae 15–24 pairs, < 2 cm wide	***B. angustipinna***
–	Sterile lateral pinnae <10 pairs, > 2 cm wide	**7**
7	The space between two secondary veins in sterile pinnae more than 7 mm wide	***B. subcordata***
–	The space between two secondary veins in sterile pinna 3–6 mm wide	***B. heteroclita* × *B. subcordata***
8	Sterile terminal pinnae having extremely elongated apex	***B. heteroclita***
–	Sterile terminal pinnae similar to lateral pinnae	**9**
9	Sterile pinnae < 3 cm wide	***B. scalpturata***
–	Sterile pinnae > 3 cm wide	***B. lianhuachihensis***

## Supplementary Material

XML Treatment for Bolbitis lianhuachihensis

## Appendix 1

**Voucher specimens and GenBank accession numbers for DNA sequences used in this study. Information is presented in the following order: species, voucher, locality, GenBank numbers for *rps4* and *trnL-F* , herbarium. “*”Newly published sequences in this study; “–“ no data.**

***Bolbitis acrostichoides***, *Faden s.n.*, Uganda, GU376645, GU376501, US. ***B. aliena***, *Jimenez 2634*, Bolivia, GU376646, GU376502, NY. ***B. angustipinna***, *Huang 170*, Taiwan, MK639320*, MK978743*, TAIF. ***B. appendiculata***, *Huang 0112*, Taiwan, MK639321*, MK978743*, TAIF. ***B. auriculata***, *Rouhan 183*, Mauritius, GU376650, AY536367, P. ***B. bipinnatifida***, *Rouhan 155*, Seychelles, GU376676, GU376530, P. ***B. crispatula***, *Wu et al. 361*, Laos, GU376655, –, MO. ***B. fluviatilis***, *Carvalho 6457*, Equatorial Guinea, GU376656, GU376510, US. ***B. gemmifera***, *Fay 1001*, Sierra Leone, GU376657, GU376511, US. ***B. heteroclita***, *Huang 140*, Taiwan, MK639322*, MK978740*, TAIF. ***B. heudelotii***, *Fay 1167*, Sierra Leone, GU376662, GU376515, NY. ***B. humblotii***, *Razafitsalana & Torge 115*, Madagascar, GU376663, GU376516, NY. ***B. lonchophora***, *Motley 2669*, French Polynesia, GU376664, GU376517, NY. ***B. major***, *Fraser-Jenkins 1811*, Nepal, GU376665, GU376518, US. ***B. portoricensis***, *McVaugh 18983*, Mexico, GU376670, GU376523, NY. ***B. rhizophylla***, *Huang 169*, Taiwan, MK639323*, MK978742*, TAIF. ***B. salicina***, *Fay 1185*, Sierra Leone, GU376671, GU376525, US. ***B. scalpturata***, *Huang 164*, Taiwan, MK639324*, MK978741*, TAIF. ***B. semipinnatifida***, *Steyermark 89173*, Venezuela, GU376672, GU376526, US. ***B. serratifolia***, *Gutierrez 1014*, Bolivia, GU376673, GU376527, NY. ***B. sinuata***, *Hoshizaki 1719*, USA, GU376675, GU376529, NY. ***B. subcordata***, *Huang 106*, Taiwan, MK639325*, MK978736*, TAIF. ***B. tibetica***, *Fraser-Jenkins 1782*, Nepal, GU376677, GU376531, US. ***B. lianhuachihensis***, *Chao 3006*, Taiwan, MK978746*, MK978747*, TAIF; *Huang 115*, Taiwan, MK639319*, MK978738*, TAIF; *Y.-F. Huang 116*, Taiwan, MK639318*, MK978737*, TAIF; *Huang 172*, Taiwan, MK639317*, MK978744*, TAIF. **B. virens var. virens**, *Lu 19796*, China, MK639326*, MK978745*, TAIF. ***Elaphoglossum lepervanchii***, *Rakotondrainibe 6359*, Madagascar, AY540228, AY536323, P. ***Teratophyllum koordersii***, *Price 981*, Philippines, GU376715, GU376566, US.

## Supplementary materials

Supplementary material 1 Figures S1–S4. Data type: species data Explanation note: **Figure S1.** Holotype of *Bolbitis lianhuachihensis* at TAIF (Y.-S Chao 3006). **Figure S2** Holotype of *Bolbitis hainanensis* at PE (C. Wang 35870). **Figure S3.** Holotype specimen of Bolbitis virens var. compacta at L (*Tagawa et al. 6802*). **Figure S4.** Holotype of *Bolbitis virens* at K (*Wallich 1033*). https://binary.pensoft.net/file/331244This dataset is made available under the Open Database License (http://opendatacommons.org/licenses/odbl/1.0/). The Open Database License (ODbL) is a license agreement intended to allow users to freely share, modify, and use this Dataset while maintaining this same freedom for others, provided that the original source and author(s) are credited.Yi-Shan Chao, Yu-Fang Huang, Shi-Yong Dong, Yao-Moan Huang, Ho-Yih Liu

## References

[B1] Chen C-W Sundue M Kuo L-Y Teng W-C Huang Y-M (2017) Phylogenetic analyses place the monotypic *Dryopolystichum* within Lomariopsidaceae. PhytoKeys 78: 83–107. https://doi.org/10.3897/phytokeys.78.12040 10.3897/phytokeys.78.12040 PMC5543276 28781553

[B2] Ching RC Wang CH (1983) Materiae ad floram filicum Sinensium. Zhiwu Fenlei Xuebao 21: 211–218.

[B3] Dong S Zhang X (2005) A taxonomic revision of the fern genus *Bolbitis* (Bolbitidaceae) from China. Zhiwu Fenlei Xuebao 43(2): 97–115. https://doi.org/10.1360/aps030105

[B4] Doyle JJ Doyle JL (1990) A rapid total DNA preparation procedure for fresh plant tissue. Focus 12: 13–15.

[B5] Hall TA (1999) BioEdit: A user-friendly biological sequence alignment editor and analysis program for Windows 95/98/NT. Nucleic Acids Symposium Series 41: 95–98.

[B6] Hennipman E (1977) A Monograph of the Fern Genus *Bolbitis* (Lomariopsidaceae). Leiden University Press, Leiden, 329 pp.

[B7] IUCN (2017) Guidelines for using the IUCN red list categories and criteria. version 13. Standards and Petitions Subcommittee, Gland and Cambridge, 108 pp. https://cmsdocs.s3.amazonaws.com/RedListGuidelines.pdf [accessed 07.14.2019]

[B8] Iwatsuki K (1959) Taxonomic studies of Pteridophyta IV. Acta Phytotaxonomica et Geobotanica 18: 44–59.

[B9] Knapp R (2011) Ferns and Fern Allies of Taiwan. KBCC Press, Pingtung and Yuan-Liou Pub, Taipei, 1052 pp.

[B10] Knapp R (2013) Ferns and Fern Allies of Taiwan – Supplement. KBCC Press, Pingtung, 212 pp.

[B11] Kuo C-M (1990) Materials for the Lomariopsidaceae of Taiwan. Botanical Bulletin of Academia Sinica 31: 305–314.

[B12] Lellinger DB (2002) A Modern Multilingual Glossary for Taxonomic Pteridology. American Fern Society, Washinton, 263 pp. https://doi.org/10.5962/bhl.title.124209

[B13] Moran RC Labiak PH Sundue M (2010a) Phylogeny and character evolution of the Bolbitidoid ferns (Dryopteridaceae). International Journal of Plant Sciences 171(5): 547–559. https://doi.org/10.1086/652191

[B14] Moran RC Labiak PH Sundue M (2010b) Synopsis of *Mickelia*, a newly recognized genus of bolbitidoid ferns (Dryopteridaceae). Brittonia 62(4): 337–356. https://doi.org/10.1007/s12228-010-9158-9

[B15] PPGI (2016) A community-derived classification for extant lycophytes and ferns. Journal of Systematics and Evolution 54(6): 563–603. https://doi.org/10.1111/jse.12229

[B16] Skog JE Mickel JT Moran RC Volovsek M Zimmer EA (2004) Molecular studies of representative species in the fern genus *Elaphoglossum* (Dryopteridaceae) based on cpDNA sequences *rbcL*, *trnL-F*, and *rps4-trnS.* International Journal of Plant Sciences 165(6): 1063–1075. https://doi.org/10.1086/423877

[B17] Souza-Chies TT Bittar G Nadot S Carter L Besin E Lejeune B (1997) Phylogenetic analysis of Iridaceae with parsimony and distance methods using the plastid gene *rps4.* Plant Systematics and Evolution 204(1–2): 109–123. https://doi.org/10.1007/BF00982535

[B18] Swofford DL (2002) PAUP*: Phylogenetic Analysis Using Parsimony (*and Other Methods), version 4. Sinauer, Sunderland.

[B19] Thiers B (2019) Index herbariorum: a global directory of public herbaria and associated staff. New York Botanical Garden’s Virtual Herbarium. http://sweetgum.nybg.org/science/ih/ [accessed 2019]

[B20] Taberlet P Gielly L Pautou G Bouvet J (1991) Universal primers for amplification of three non-coding regions of chloroplast DNA. Plant Molecular Biology 17(5): 1105–1109. https://doi.org/10.1007/BF00037152 1932684 10.1007/BF00037152

[B21] Thompson JD Higgins DG Gibson TJ (1994) CLUSTAL W, improving the sensitivity of progressive multiple sequence alignment through sequence weighting, position-specific gap penalties and weight matrix choice. Nucleic Acids Research 22(22): 4673–4680. https://doi.org/10.1093/nar/22.22.4673 7984417 10.1093/nar/22.22.4673 PMC308517

[B22] Tsai J-L Shieh W-C (1994) Lomariopsidaceae. In: Huang T-C Peng C-I Boufford D-E Hsieh C-F Lowry PPI Ohashi H (Eds) Flora of Taiwan.Editorial Committee of Flora of Taiwan, second Edition, Taipei, 352–363.

[B23] Zhang L-B Wu S-G Xiang J-Y Xing F-W He H Wang F-G Lu S-G Dong S-Y Barrington D Iwatsuki K Christenhusz MJM Mickel JT Kato M Gilbert MG (2013) Dryopteridaceae. In: Wu ZY Raven PH Hong DY (Eds) Flora of China.Science Press, Beijing; Missouri Botanical Garden Press, St. Louis, 542–724.

[B24] Zwickl DJ (2006) Genetic Algorithm Approaches for the Phylogenetic Analysis of Large Biological Sequence Datasets under the Maximum Likelihood Criterion. PhD Thesys, The University of Texas, Austin.

